# Wound healing effects of dexpanthenol-loaded core/shell electrospun nanofibers: Implication of oxidative stress in wound healing

**DOI:** 10.22038/IJBMS.2023.71412.15526

**Published:** 2024

**Authors:** Seyede Sahar Hashemi, Mahmoud Najari, Milad Parvin, Mohammad Mehdi Kalani, Majid Assadi, Ramin Seyedian, Sasan Zaeri

**Affiliations:** 1 Student Research Committee, Bushehr University of Medical Sciences, Bushehr, Iran; 2 Department of Oral & Maxillofacial Surgery, School of Dentistry, Bushehr University of Medical Sciences, Bushehr, Iran; 3 Department of Life Science Engineering, Faculty of New Sciences and Technologies, University of Tehran, Tehran, Iran; 4 Nuclear Medicine and Molecular Imaging Research Center, Bushehr University of Medical Sciences, Bushehr, Iran; 5 Department of Pharmacology, School of Medicine, Bushehr University of Medical Sciences, Bushehr, Iran

**Keywords:** Core/shell nanofiber, Dexpanthenol, Fibroblast, Mouse, Oxidative stress, Wound healing

## Abstract

**Objective(s):**

Knowing the detrimental role of oxidative stress in wound healing and the anti-oxidant properties of Dexpanthenol (Dex), we aimed to produce Dex-loaded electrospun core/shell nanofibers for wound healing study. The novelty was measuring oxidative stress in wounds to know how oxidative stress was affected by Dex-loaded fibers.

**Materials and Methods:**

TPVA solution containing Dex 6% (w/v) (core) and PVA/chitosan solution (shell) were coaxially electrospun with variable injection rates of the shell solution. Fibers were then tested for physicochemical properties, drug release profile, and effects on wound healing. Levels of tissue lipid peroxidation and superoxide dismutase activity were measured.

**Results:**

Fibers produced at shell injection rate of 0.3 ml/hr (F3 fibers) showed core/shell structure with an average diameter of 252 nm, high hydrophilicity (swelling: 157% at equilibrium), and low weight loss (13.6%). Dex release from F3 fibers seemed to be ruled by the Fickian mechanism based on the Korsmeyer-Peppas model (R^2^ = 0.94, n = 0.37). Dex-loaded F3 fibers promoted fibroblast viability (128.4%) significantly on day 5 and also accelerated wound healing compared to the neat F3 fibers at macroscopic and microscopic levels on day 14 post-wounding. The important finding was a significant decrease in malondialdehyde (0.39 nmol/ mg protein) level and an increase in superoxide dismutase (5.29 unit/mg protein) activity in Dex-loaded F3 fiber-treated wound tissues.

**Conclusion:**

Dex-loaded core/shell fibers provided nano-scale scaffolds with sustained release profile that significantly lowered tissue oxidative stress. This finding pointed to the importance of lowering oxidative stress to achieve proper wound healing.

## Introduction

In the domain of skin care and dermatology, skin wound healing following injuries, burns, diabetic wounds and other insults to intact skin has always been one of the main concerns. It is proposed that choosing an appropriate wound healing agent or wound dressing is of paramount importance as it has a significant impact on the restoration of the skin tissue, the mechanical properties of the skin, and the prevention of dermal invasions by microorganisms. Also, the aesthetic aspect of wound healing is significantly affected by the healing agent or dressing.

Oxidative stress plays a major role in the fate of wound healing. During wound healing, wounded tissue undergoes four consecutive phases: hemostasis, inflammation, proliferation, and maturation. Reactive oxygen species (ROS) naturally rise at the inflammatory phase to help the immune system against invading pathogens and also to stimulate early angiogenesis and cell proliferation. However, uncontrolled levels of ROS will eventually act as a complicating factor in the next phases of wound healing. For example, ROS have been shown to delay diabetic wound healing (1). Also, elevation of ROS destroys collagen fibers by enhancing the expression of matrix metalloproteinases (2). Therefore, using anti-oxidant compounds to target oxidative stress in wound tissue may be considered a logical approach to achieving proper wound healing.

Dexpanthenol (Dex) is one of the healing enhancers with prominent anti-oxidant activity. It is an alcoholic derivative of vitamin B_5_ and is present in a variety of cosmetics and skin-related formulations due to its proven dermato-protective activities (3). Previous studies have reported prominent anti-oxidant activity for Dex. For example, the administration of Dex to diabetic rats led to significantly lower malondialdehyde (MDA) and higher superoxide dismutase activity in the kidney tissue compared to the control group (4). In addition, Dex significantly reduced oxidative stress in animal models of neuropathy (5) and liver injury (6). Hence, we selected Dex as the active pharmaceutical with anti-oxidant properties in the present study on wound healing.

In order to achieve proper wound healing and to prevent any healing complications, a wide range of topical formulations including ointments, gels, creams, and lotions are usually used. However, such traditional pharmaceuticals have a number of shortcomings. For instance, the patient has to frequently apply these topicals on the wound because there is generally no mechanism for controlled release of the drug in these simple topical formulations. Furthermore, these formulations may fail to provide enough contact between the wound-healing drug and the wound medium. Consequently, the probability of active interactions between cells and macromolecules in the wound environment with the contained agent(s) would be minimized (7). To resolve these shortcomings, nanofibers have recently been introduced as new drug delivery systems. They can be loaded with active pharmaceuticals to produce new wound dressings that possess several advantages over conventional topical formulations. For example, nanofibers highly resemble the skin’s natural extracellular matrix (8). Additionally, they have high surface-to-volume ratios, high porosity, biological degradability, appropriate attachment to cells, and the ability to be adjusted for drug release (9, 10). Nanofibers may be made up of different materials either with synthetic (polyvinyl alcohol (PVA), polylactic acid, etc.) or natural (chitosan, collagen, etc.) origin (11). In recent years, wound dressings with nanofibrous structures have been mainly fabricated by means of the electrospinning technique. In electrospinning, a polymer solution that might contain one or more ingredients is subjected to a very high electrical voltage which makes the polymer solution stretch strongly in the electrical field. This process is accompanied by the evaporation of the solvent and production of fine non-woven fibers at the nano-scale level. The produced nanofibers can provide a good platform for effective interaction with fibroblasts, macrophages, and other important moieties in the wound-healing environment (12).

Coaxial electrospinning is an interesting version of the common electrospinning method and is widely used to produce nanofibers with a core/shell structure. Briefly, in coaxial electrospinning, the needle has two coaxial channels: one for the core solution (internal channel) and the other for the shell solution (external channel). In this method, the main purpose is to load the bioactive pharmaceutical into the core while protecting it with an outer shell layer. This would ensure the control of drug release and the preservation of the drug in the internal core against external challenges such as decomposition by light, oxidation, etc. In order to enhance wound healing, the shell layer can be incorporated with bioactive materials to add further biological features to the fibers (13). 

Loading Dex in the core of a core/shell nanofiber may be a promising approach to control its release to the wound medium. On the other hand, the composition of the shell layer can be enriched by a polymer with a bioactive wound healing agent. Accordingly, chitosan (CS) was chosen to be incorporated in the shell layer in the current study; CS is an amine-bearing polysaccharide and has antimicrobial, anti-bleeding, anti-inflammatory, and wound-healing properties (14, 15).

**Figure 1:**
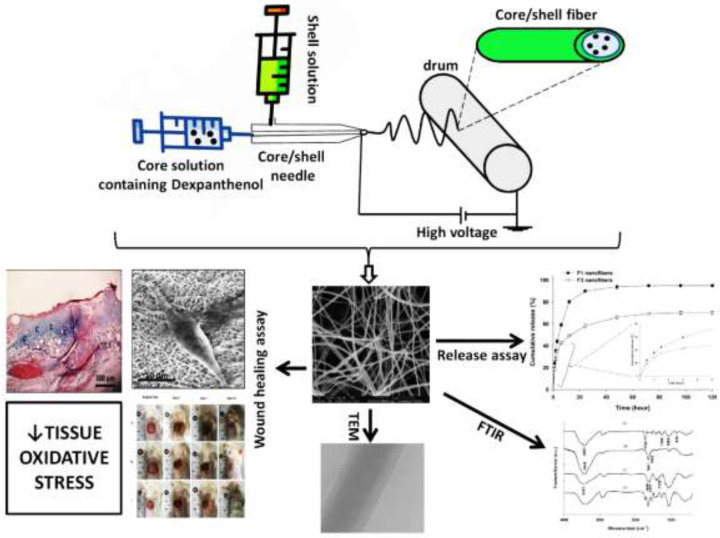
Outline of the study consisting of core/shell fiber production, characterization, and wound healing assays

Knowing the novelty of the nanofibrous wound dressings with core/shell structure and the promising wound healing and anti-oxidant potential of Dex, we aimed to design and fabricate a simple core-shell nanofibrous wound dressing loaded with Dex in the core. Accordingly, physicochemical properties, *in vitro* drug release kinetics, *in vitro* effects on human fibroblasts, and *in vivo* wound healing activity of the fabricated nanofibers were evaluated. One important novelty compared to the previous works is that the probable wound-healing mechanism of Dex-loaded fibers has been explored by measuring oxidative stress parameters in wound tissues. Figure 1 illustrates the outline of the study design and findings.

## Materials and Methods

***Materials***

Dexpanthenol (Dex) powder was provided by Oxin Darou Vesht Co. (Iran). Polyvinyl alcohol (PVA) (MW 72,000 Da) was purchased from Merck Co. (Germany). Chitosan (MW 600,000 Da, deacetylation degree 76%), and glutaraldehyde (25% vol/vol) were ordered from Fluka Co. (UK). Vials of Ketamine and xylazine were supplied from Alfasan Co. (the Netherlands). Double distilled water (dd H_2_O) was homemade and served as diluting solvent when needed throughout the experiments.

***Electrospinning of solutions***

A solution containing PVA (5% wt/vol) and Dex (6% wt/vol) was used as the core solution. Briefly, PVA powder was dissolved in hot dd H_2_O (80°C) by stirring for 3 hr. After the solution was cooled to room temperature, Dex powder was added to reach 6% (wt/vol) concentration. A solution containing PVA-CS at 4.5%-0.5% (wt/vol) was used as the shell solution. Briefly, CS was first dissolved in an acetic acid (0.1 M) solution and then added to the PVA solution to reach the above concentration in the shell solution. Electrospinning was performed using an electrospinning apparatus supplied by Fanavaran Nano Meghyas Co. (Iran). Table 1 summarizes electrospinning parameters for fiber production. The injection speed of the shell solution was chosen as the variable parameter at three levels. Accordingly, three fibers (F_1_, F_2_, and F_3_) were produced each at the defined injection rate of the shell solution. Fibrous mats were finally exposed to a vapor of a diluted glutaraldehyde solution (10% vol/vol) for 12 hr at ambient temperature so that fibers could be cross-linked. This step was necessary to prevent rapid disintegration of the wound dressings in the aqueous medium. Subsequently, the mats were gently rinsed in dd H_2_O to clear unreacted glutaraldehyde and were desiccated in a vacuum.

***Diameter and morphology of fibers***

Fiber size and morphology assessment was performed using Scanning electron microscopy (SEM) (CamScan-MV2300, USA). In short, small pieces of mats were coated with gold particles and exposed to high voltage (20 kV). Determination of fiber diameters was done in ImageJ Software v. 1.47 (National Institutes of Health, USA). The mean diameter of fiber in each mat was calculated by measuring diameters of 50 to 100 random fibers in the corresponding SEM image. Further, to show the core/shell structure of the fabricated fibers, transmission electron microscopy (TEM) (Philips CM30, the Netherlands) was used.

***Swelling and weight loss tests***

To conduct the swelling test, the mats were inserted into phosphate-buffered saline (PBS) (pH 7.4, 25°C) for up to 24 hr. Equation 1 was used to calculate the swelling (%) of mats: 

$\mathrm{Swelling ratio}\left(\%\right)=\frac{M_{t}-M_{i}}{M_{i}}\times 100$

*Equation 1*

*M*_i_ *is the mat weight before insertion into the PBS buffer.* *M*_t_ is its weight after insertion into the buffer at time ***t***. The time at which no more swelling occurred (see Results, Figure 3) was selected as the swelling equilibrium time and the corresponding data were chosen to be compared among mats. 

In the weight loss test, a small amount of mat (~50 mg) was weighed (*Mi*), immersed in PBS (20 ml, pH 7.4, 37 °C), and left for 72 hr. Then, it was dried and weighed again (*M*_72_). Percent weight loss (%) was reported using Equation 2. 

$\mathrm{Weight loss}\left(\%\right)=\frac{M_{i}-M_{72}}{\mathrm{Mi}}\times 100$

Equation 2

***Fourier transform infrared spectroscopy (FTIR)***

The chemical identity of the electrospun fibers and probable chemical interaction(s) between the components in fibers (PVA, Dex, and CS) were assessed using FTIR (Perkin Elmer, Frontier, USA). In order to obtain the FTIR spectra, PVA powder, Dex powder, CS powder, and the electrospun mat containing all the above ingredients each was separately mixed with potassium bromide at 1:100 (wt/wt). The FTIR spectra covered a range of 400 to 4000 cm^-1^. 

***Dexpanthenol release test***

The F_1_ (Dex-loaded core fiber) and F_3_ (Dex-loaded core/shell fiber) fibers were chosen for the release study. They were separately put in the PBS solution (15 ml, pH = 7.4, 37 °C) under mild stirring for 120 hr. At different intervals, i.e., 1, 2, 3, 6, 12, 24, 72, 96, and 120 hr, 2 ml of the solution were sampled to determine Dex concentration spectrophotometrically (Shimadzu UV-1100) at 210 nm. A calibration curve for Dex (0-100 µg/ml) was created to calculate drug concentration in the release medium. To measure the absorbance of Dex in serial dilutions (calibration curve) and test samples, we tried to zero any absorbance of materials other than Dex by using a blank solution that contained a piece of neat PVA/chitosan mat. The blank was treated the same way as the release-test samples to allow any possible dissolution of PVA or chitosan into the buffer medium. This would ensure that the final reported absorbance at 210 nm belonged to Dex itself. An equivalent (2 ml) volume of PBS solution was added back to the release solution to compensate for the lost volume sampled at each interval. The mechanism of Dex release was also studied using zero order, first order, Higuchi, and Korsmeyer–Peppas models (See Results section, Table 2) (16). 

***In vitro cell attachment and viability test***

To show nanofiber performance on cell attachment and viability, human fibroblasts (HNFF-P18 cell line, Pasteur Institute Cell Bank, Iran) were cultured onto nanofibers and then underwent the 3-(4, 5-dimethyl-2-thiazolyl)-2, 5-diphenyl-2H-tetrazolium bromide (MTT) assay. First, the HNFF-P18 cells were incubated under 5% CO_2_ at 37 °C in the presence of DMEM medium which was enriched with 10% (v/v) fetal bovine serum, penicillin (100 U/ml) and streptomycin (100 μg/ml). The cells were subsequently seeded into nanofiber-coated wells in a 24-well multi-plate at a density of 2.5 ×10^4^ cells per well. Then, they were incubated for 1, 3, and 5 days. The MTT solution was added to each well and the cells were incubated for an extra 4 hr. Finally, absorbance in each well was read at 570 nm using a microplate reader (Power waveX, Bio-tek Instruments, USA). Fibroblast viability was calculated using Equation 3 in which *A*_s_, *A*_c_, and *A*_b_ indicate absorbance of the sample (mat-treated fibroblasts), control (neat fibroblasts), and blank, respectively. 

$\mathrm{Fibroblast viability}\left(\%\right)=\frac{A_{s}-A_{b}}{A_{c}-A_{b}}\times 100$

***Animal wound healing study***

*Animals and wound model*

Male albino mice (25–30 g) aged 2.5 to 3 months were chosen for the wound healing study. Animals were purchased from the laboratory animal breeding center of Bushehr University of Medical Sciences (BPUMS), Iran. All animals were transported to the working place one week before the onset of experiments for familiarization purposes. They were placed in standard cages each containing 3 animals with free access to drinking water and pellet chow. To maintain their diurnal life pattern, 12-hr light-dark cycles were provided through the experiments. The animal study received an allowance from the Ethics Committee of BPUMS (IR.BPUMS.REC.1396.200), and animals were treated based on the ethical guidelines for the use of animals in research.

A circular full-thickness excisional wound (1 cm in diameter) was created on the back (interscapular region) of each mouse under intraperitoneal general anesthesia with a mixture of ketamine (60 mg/kg) and xylazine (10 mg/kg). Before wounding, the area was well shaved and disinfected with 70% ethanol. Wounding was performed with a pair of sharp curved scissors. Attempts were made to make wounds with smooth edges and not to nick the subcutaneous tissues.

*Experimental design*

A total of 18 mice were assigned to three experimental groups (n = 6 mice/group) to be topically treated with one of the followings: normal saline (group 1), neat F_3_ fibers [without Dex in the core] (group 2) and Dex-loaded F_3_ fibers [with Dex in the core] (group 3). As would be presented later, F_3_ fiber demonstrated superior physicochemical characteristics over F_1_ and F_2_ fibers and hence was selected for the wound healing study. Animals in each group received daily treatments for 14 days. Wounds were photographed on days 0 (surgical day), 3, 7, and 14 post-surgery. Wound photos were then inserted into the ImageJ Software v. 1.47 to calculate the wound area. The following equation was used to report the wound area:

In this formula, *A*_0_ is the initial wound area (day zero) and *A*_d_ is the wound area on the selected day. On days 7 and 14 post-surgery, wound tissue was carefully excised and divided into two parts. One part was preserved in formalin for histopathology study and the other part was stored at ­80 °C to be used for oxidative stress test. For the histopathology study, tissues were processed and stained with hematoxylin–eosin (H&E) and Masson’s trichrome under the guidance of routine protocols. Re-epithelization, granulation tissue, and collagen density in wounds were evaluated and reported qualitatively in terms of extent or severity (absent, mild, moderate, and complete or abundant) by an expert pathologist blind to the experimental groups.

*Measurement of oxidative stress parameters*

The state of oxidative stress in terms of levels of lipid peroxidation and superoxide dismutase (SOD) activity in wound tissues was evaluated. First, tissues were unfrozen, homogenized in PBS buffer (pH = 7.4, 4 °C), and centrifuged at 4000 rpm. The total protein content in the supernatant was measured using the Bradford method (17). To determine lipid peroxidation, the level of malondialdehyde (MDA) (nmol/mg total protein) which is the main by-product of lipid peroxidation was measured using spectrophotometry at 532 nm. To determine the SOD activity (unit/mg of total protein) which is a measure of cellular anti-oxidant defense, the absorption of a product obtained from the reaction between superoxide anions and a dye was measured at 440 nm. When SOD activity is high, fewer superoxide anions are present in cells, and hence less absorption will be obtained. To conduct both the aforementioned assays, kits of MDA and SOD assays were used under the guidance of the company’s instructions (Abnova, Taiwan).

***Statistical analysis***

All presented data were collected through at least three repeated experiments (mean ± standard deviation). Data of cell viability in the MTT assay were analyzed by independent samples T-test. Data of wound areas and biochemical measurements (MDA and SOD) were analyzed by one-way ANOVA followed by Tukey *post hoc* test. Statistical tests were performed in SPSS^®^ Software (v.21) and the differences were defined as significant at *P*-values less than 0.05. 

## Results

***Morphology, diameter, and structure of fibers***

**Figure 2:**
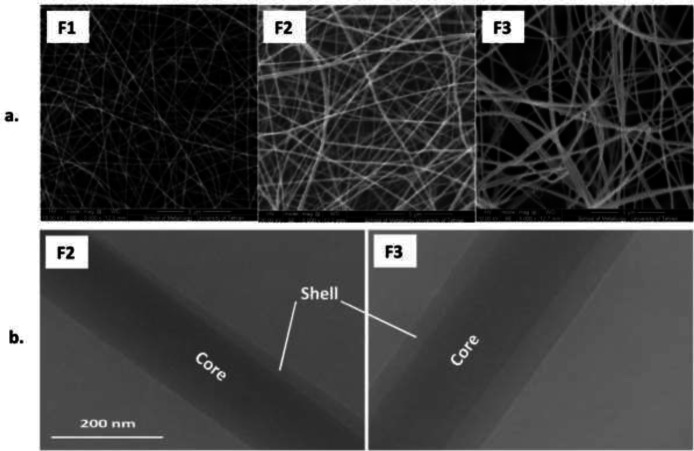
(a.) Representative SEM photos of F1, F2, and F3 fibers electrospun at shell solution injection rates of 0, 0.15, and 0.3 ml/hr, respectively. (b.) Representative TEM images of F2 and F3 fibers

SEM images of F_1_, F_2_, and F_3_ fibers are shown in Figure 2a. The mean diameters of fibers in F_1_, F_2_, and F_3_ mats were 167.6 ± 31.4, 208.3 ± 57.8, and 252.0 ± 70.7 nm, respectively. This finding shows that by increasing the flow rate of the shell solution from 0 ml/hr to 0.3 ml/hr an increase in the thickness of the shell and thus in the overall diameter of the fibers was obtained. Figure 2b shows representative TEM images of F_2_ and F_3_ core/shell fibers (the shell solution was not used in the F_1_ nanofiber, so this nanofiber had no shell, and therefore TEM imaging was not performed). Although the core-shell structure was formed in the F_2_ fibers, the shell layer was not formed completely around the core. This might be attributable to the relatively lower injection rate of the shell solution (0.15 ml/hr). Instead, when the injection rate of the shell solution increased (0.3 ml/hr), the shell layer was formed uniformly around the core. Therefore, the results of TEM imaging showed that fibers were produced with a distinct core-shell structure when the shell solution (PVA/CS) was injected at a rate of 0.3 ml/hr.

***Swelling and weight loss tests***

**Figure 3:**
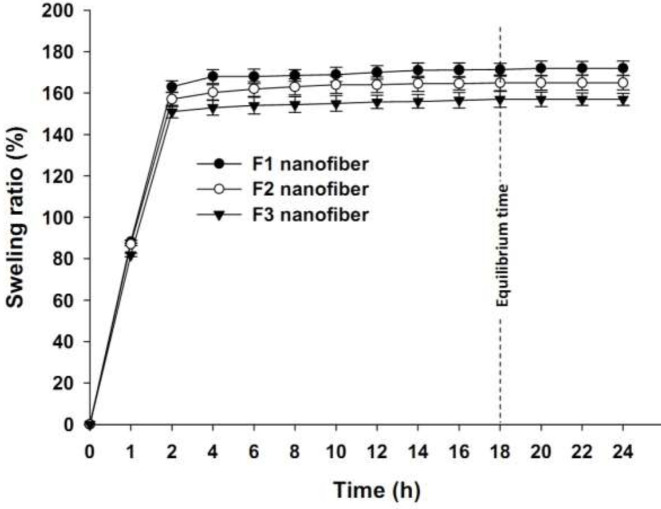
Swelling ratios (mean ± SD) of F1, F2, and F3 nanofibers at different time intervals

Swelling ratio and weight loss tests were used as measures for hydrophilicity and resistance against the disintegration of fibers, respectively. At the equilibrium, fibers swelled up to 157-172% depending on the fiber type (Figure 3). The F_3_ fiber presented a lower swelling ratio compared to its F_1_ and F_2_ counterparts. Findings of the weight loss test revealed that F_3_ fibers lost almost less weight (13.6% ± 0.3) than F_1_ (14.5% ± 0.4) or F_2_ fibers (14.2% ± 0.4) meaning that F_3_ fibers were nearly more resistant against disintegration in the PBS buffer after 72 hr.

***Fourier transform infrared spectroscopy (FTIR) analysis***

**Figure 4:**
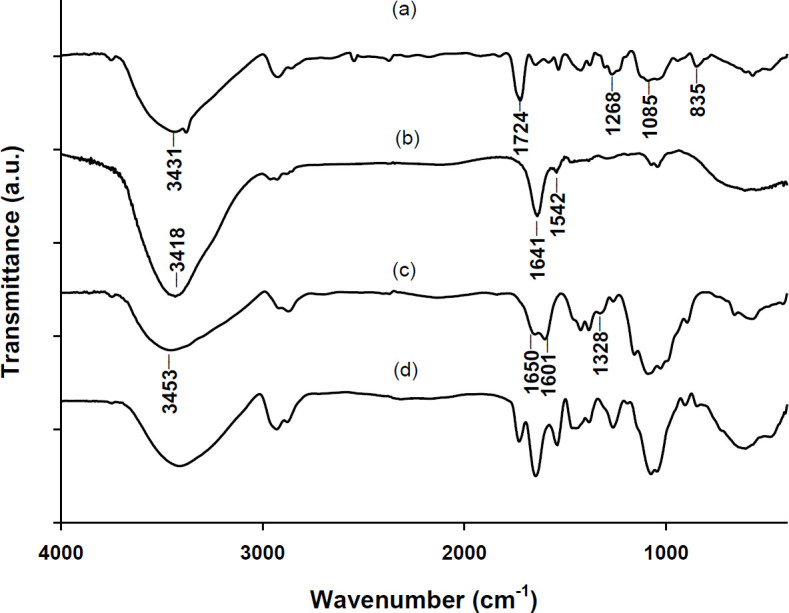
FTIR spectra of (a) pure PVA, (b) pure Dex, (c) pure CS, and (d) Dex-loaded core/shell electrospun fibers (F3)

The FTIR spectra of pure PVA, Dex, and CS powders as well as the final product, i.e., Dex-loaded core/shell nanofiber (F_3_) are shown in Figure 4a-4d. The wide peak at 3431 cm^-1^ in the PVA spectrum (Figure 4a) is due to the stretching vibration of the hydroxyl groups (-OH). The peaks at 835, 1085, and 1268 cm^-1^ can be attributed to the C-C stretching, C-O stretching, and C-H bending vibrations, respectively (18). The sharp band at 1724 cm^-1^ was probably due to the presence of residual acetyl groups (C-C=O) in the PVA polymer. In the Dex spectrum (Figure 4b), the distinct wide band at 3418 cm^-1^ is attributed to the presence of hydroxyl (-OH) groups, and the peaks at 1641 and 1542 cm^-1^ are due to the stretching of the carbonyl group (amide I) and bending of N-H group (amide II) in the Dex molecule. Similar characteristics peaks were reported for Dex previously (19, 20). In the CS spectrum (Figure 4c), the wide peak centered at 3453 cm^-1^ belongs to the stretching vibration of O-H and N-H groups and the intramolecular hydrogen bonds. The peaks at 1650 cm^-1^ (C=O stretching of amide I) and 1328 cm^-1^ (C-N stretching of amide III) represent N-acetyl residual in CS. The presence of primary amine (N-H bending) in CS was confirmed by the band at 1601 cm^-1 ^(21). The FTIR spectrum of F_3_ fibers (Figure 4d) contained almost all the aforementioned diagnostic bands of the pure components without any extra bands or band shifts confirming that no significant chemical interaction occurred between PVA, CS, and the drug, and that the electrospinning process did not adversely affect chemical entity of the primary materials.

***Dexpanthenol release test***

**Figure 5:**
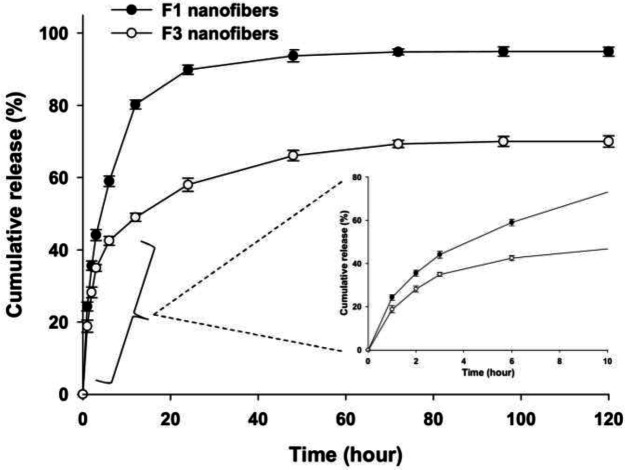
Cumulative release profile of Dex from F1 and F3 fibers in the PBS buffer solution (pH = 7.4, 37 °C)

F_3_ fiber exhibited a distinct core/shell structure as shown in the TEM image, and hence was selected for the drug release test to be compared with the shell-less fiber (F_1_). Dex release from F_3_ fiber was slower than its F1 counterpart within the 120-hr time span. While the final cumulative release percentage of Dex from the F_1_ fibers reached 94%, this value was about 70% for the F_3_ fibers (Figure 5). Such differences were quite expectable because the shell layer in the core/shell F_3_ fibers is thought to act as a barrier against Dex release. 

In order to propose a mechanism for Dex release from the fabricated fibers, a number of release kinetic models were used to fit the release data (Table 2). In the case of F_1_ fibers, data were best fitted into the Higuchi and Korsmeyer-Peppas models with R^2^ values of 0.99. Similar results were obtained for the F_3_ fibers where the best regressions were found in the Higuchi (R^2^ = 0.93) and Korsmeyer-Peppas (R^2^ = 0.94) models. The n value which determines the release mechanism in the Korsmeyer-Peppas model was found to be 0.49 for the F_1_ and 0.37 for the F_3_ fibers.

***Compatibility of F***_3_ ***fibers with cultured human fibroblasts***

**Figure 6:**
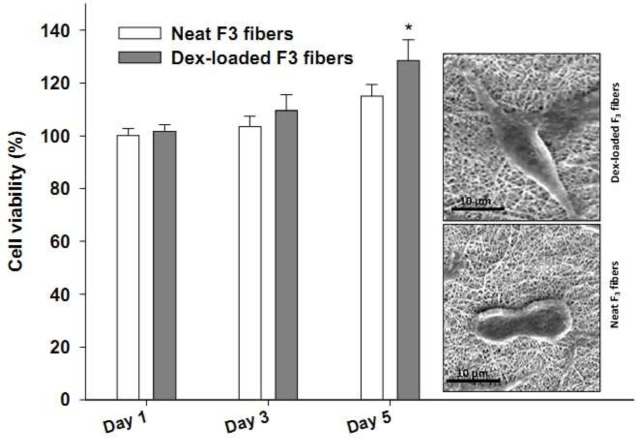
Percent viability (mean ± standard deviation, n = 5) of the human fibroblasts grown on Dex-loaded or neat F3 fibers for 1, 3, and 5 days

Due to the convincing physicochemical properties of the core/shell F_3_ fibers and their better control over Dex release as compared to their F_1_ counterpart, the F_3_ fibers were chosen for the biological assays including *in vitro *and *in vivo* wound healing evaluations. Figure 6 demonstrates the effects of F_3_ fibers, loaded with or without Dex, on fibroblast viability and morphology. The results showed that viability values were all above 100% for cells treated with F_3_ fibers either with or without Dex within a 5-day period. This clearly indicates the biocompatibility of the fabricated fibers. By comparing viabilities between drug-loaded and neat F_3_ fibers on each selected day, it was revealed that the viability percent of Dex-loaded F_3_ fibers (128.4%) was significantly higher than that of neat F_3_ fibers (114.9%) (*P*=0.011) on day 5 of the experiment. The SEM photos of representative fibroblasts either treated with Dex-loaded F_3_ fibers or neat F_3_ fibers for 5 days were also shown in Figure 6. The Dex-loaded F3-treated fibroblast adopted a more elongated form on the fibers compared to the neat F3-treated fibroblast. 

***Findings of in vivo wound healing study and biochemical assay***

*Wound area*

**Figure 7:**
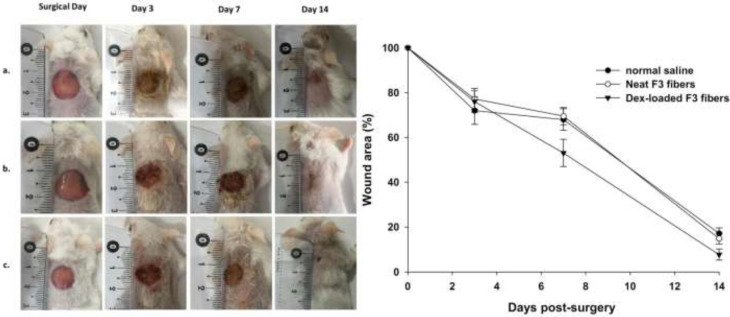
Representative photos of skin wounds treated with (a) normal saline, (b) neat F3 fibers, and (c) Dex-loaded F3 fibers at different treatment days (n = 5–6 mice per group).

Representative photos of wounds treated with normal saline, neat and Dex-loaded F_3_ fibers for a period of 14 days together with the corresponding plot of wound areas are shown in Figure 7. As this figure depicts, a decreasing pattern in wound size could be observed in each treatment group from day 0 (surgical day) to day 14. However, wounds treated with Dex-loaded F_3_ fibers showed a faster-decreasing pattern. These wounds had an average wound area of 7.7 ± 2.5% at day 14 which was the smallest wound size among others. Statistical analysis of wound sizes found significant differences between groups on day 7 (F = 17.26, df = 2, *P*<0.001) and 14 (F = 19.87, df = 2, *P*<0.001) post-surgery. Wounds treated with Dex-loaded F_3_ fibers were smaller by 21.9% and 23.5% than wounds treated with normal saline and neat F_3_ fibers, respectively on day 7. Also, they were smaller by 55.2% and 48.3% than the aforementioned groups on day 14. This means that the between-group difference in wound size was higher on day 14 than on day 7 post-surgery. 

*Wound histopathology*

**Figure 8:**
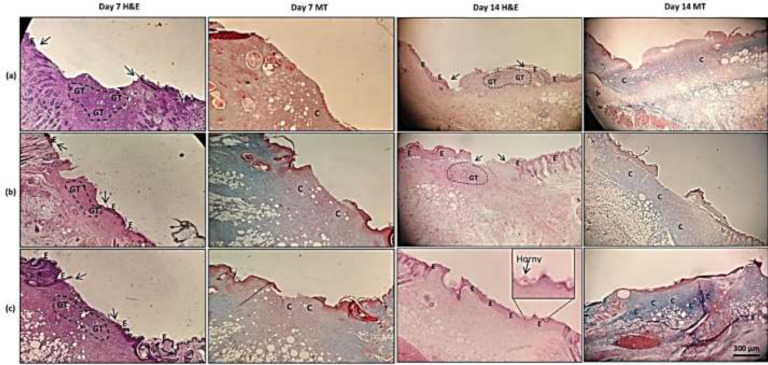
Representative photos of mice wound tissues on days 7 and 14 post-surgery stained with hematoxylin and eosin (H & E) and Masson-trichrome (MT) protocols. Treatments included (a) normal saline, (b) neat F3 fibers, and (c) Dex-loaded F3 fibers

Representative photomicrographs of wound tissues after treatment with normal saline, neat or Dex-loaded F_3_ fibers for 7 and 14 days are shown in Figure 8. Tissues were stained with hematoxylin and eosin (H&E) and Masson-trichrome (MT) protocols. Comparing tissues on day 7, all wounds showed a large amount of edema and granulation tissue, especially in the normal saline group. Granulation tissue in the healing process is inevitably formed at the first stages of wound healing and is actually necessary for proper wound healing but is not supposed to be present beyond the first few days of healing. It mainly consists of a mixture of activated fibroblasts, inflammatory cells, and cytokines in an edematous context. Re-epithelization in the normal saline group had already started but had a better progression in neat F_3_ and Dex-loaded F_3_ groups. While Masson staining showed weak collagen formation in the normal saline group, it presented a more collagenous context (blue color) in the other two groups. In MT staining, collagen fibers specifically dye blue. So, the more intensive blue color means more collagen production. After treating wounds for 14 days, those treated with normal saline presented mild (weak) re-epithelization in contrast to moderate and complete re-epithelization in wounds treated with neat F_3_ fibers and Dex-loaded F_3_ fibers, respectively. It is noteworthy that a thin horny layer (keratinization) was formed on the re-established epithelium in wounds treated with either neat or Dex-loaded F_3_ fibers. Granulation tissue was found to be moderate to high in the normal saline group, mild to moderate in the neat F_3_ fiber group, and almost scarce in the Dex-loaded F_3_ fiber group. In terms of collagen production, the most abundant collagen formation was seen in wounds that received Dex-loaded F_3_ fibers (Figure 8c). This was evidenced by a higher density of the blue color in the MT-stained wound tissues in this group compared to other treatments; Skin appendices including hair follicles were not observed in the recovered wound tissue in any of the experimental groups on day 14 post-surgery. Collectively, wounds treated with drug-loaded F_3_ fibers presented better histopathologic findings than other wounds. These findings were consistent with the results of wound area (macroscopic) measurement in which the smallest wounds belonged to the Dex-loaded F3 fiber-treated wounds.

*Findings of oxidative stress parameters in wounds*

**Figure 9:**
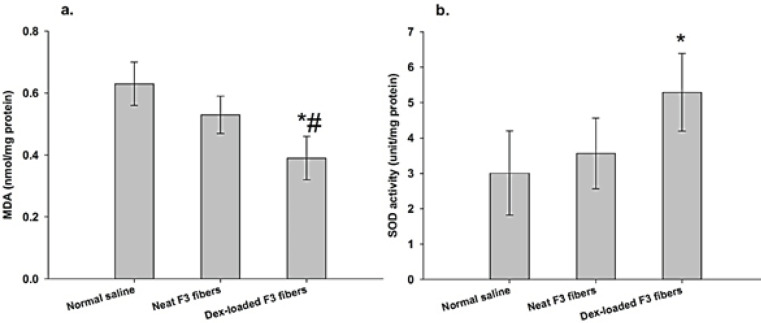
Levels (mean ± standard deviation) of oxidative stress markers (a) malondialdehyde (MDA) and (b) superoxide dismutase (SOD) activity in mice wounded tissues on day 14 (n = 5-6/group)

Levels of MDA and SOD activity as indices of tissue oxidation-reduction status on day 14 post-surgery are reported in Figure 9. Between-group comparisons of MDA data by ANOVA yielded significant differences (F =14.76, df = 2, *P*=0.001). In detail, treatment of wounds with neat F_3_ fibers reduced the MDA level (index of lipid peroxidation) by 15.8% compared to treatment with normal saline but the difference here did not reach statistical significance (*P*=0.15). Addition of Dex to the fibers’ core (Dex-loaded F_3_ fibers), however, caused a significant reduction in the MDA levels (by 38.0%) compared to normal saline (*P*=0.001). It was also revealed that tissue MDA level in the Dex-loaded F_3_ fibers group was significantly lower (by 26.4%) than that in the neat F_3_ fibers (*P*=0.026). Between-group comparisons of SOD activities also pointed to a significant difference (F = 5.74, df = 2, *P*=0.018). Based on *post hoc* analysis, there was an increase in SOD activity following the treatment of wounds with neat F_3_ fibers with reference to normal saline although the difference was not significant (*P*=0.70). Notably, the level of SOD activity in Dex-loaded F_3_ fiber-treated wounds was higher than that of normal saline-treated wounds by 75.7% (*P*=0.018).

## Discussion

Recent studies have extensively pointed to the important role of the oxidation-reduction (redox) state of wounds in the healing process (22, 23). Hence, strategies that seek moieties with effective anti-oxidant or redox-modulating properties seem to be highly promising in the area of tissue regeneration. On the other hand, wound dressings with nanofibrous texture as a novel topical drug delivery system have attracted much attention among wound-healing researchers. One of the important findings of this study was the significant effectiveness of the Dex-loaded core/shell fibers in wound closure and healing process in comparison to other treatments. Unlike previous studies on Dex-loaded nanofibrous systems (19, 24-27), we moved one step further by measuring oxidative stress parameters in the wounds. 

In the present work, fibers were electrospun co-axially using PVA-Dex in the core and PVA-CS in the shell solutions. The fabricated fibers were all bead-free and uniform in shape. The result of the study also indicated that the mean diameter of the fabricated fibers increased upon increasing the flow rate of the shell solution. This finding is consistent with the findings obtained by another study in which magnetic gold-coated core/shell nanofibers loaded with two anticancer drugs were synthesized. The authors reported that by increasing the injection rate of the polycaprolactone-polyurethane-based shell solution from 0.30 to 0.80 ml/hr, the mean fiber diameter increased from 230 nm to 340 nm (28). 

Due to its polyalcoholic structure, PVA is soluble in water and gives a hydrophilic nature to the final product, i.e., the electrospun fibrous mats. F_3_ fibers showed less swelling ratio (157%) in the swelling test compared to F_1_ (172%) and F_2_ (165%) fibers. This might be due to the presence of the uniform shell layer consisting of CS which tends to decrease the hydrophilicity of nanofiber surface as compared to the shell-less fibers (F_1_) which lacked CS on the surface or F_2_ fibers which had a thinner non-uniform shell layer. However, F_3 _fibrous mat is still considered a hydrophilic product due to its high swelling ratio value, i.e., 157% at the equilibrium time. So, this mat is highly recommended for pus-secreting or wet wounds which require water-absorbing wound dressings. 

In the drug release assay, the release curve of F_3_ fibers showed a more gradual pattern of release compared to the F_1_ fibers. Dex cumulative release from F_3_ fibers was lower than F_1_ fibers at each sampling point and finally reached ~70% of the primary loaded drug. As the primary amount of Dex in both fiber types was the same, we should seek other parameters to explain the observed difference in the release profiles of F_1_ and F_3_ mats; these may include fibers’ diameter, hydrophilicity, core/shell structure, etc. The results showed that F_1_ fibers were thinner and more hydrophilic than F_3_ fibers, so they tended to get in contact with the aqueous medium more easily to release Dex. However, the presence of the shell layer in the F_3_ fibers maybe even a more determinant factor to explain why Dex was released more gradually from the F_3_ fibers than its F_1 _counterpart. Accordingly, Yan *et al.* reported that the release of doxorubicin (an anticancer drug) from core/shell nanofibers (PVA in the core and polycaprolactone in the shell) fell when the thickness of the shell layer was increased by raising the sell solution flow rate (29). 

Investigation of the drug release mechanism revealed that the Korsmeyer-Peppas model best explained Dex release from nanofibers. In this model, n values less than 0.45 suggest the Fickian mechanism (30). So, F_3_ fibers (n = 0.37) followed Fickian release, whereas F_1_ fibers (n = 0.49) tended to release Dex by a non-Fickian mechanism. In the Fickian mechanism, drug release is determined by the amount of drug in the carrier (concentration gradient) as well as the swelling of the polymeric carrier (31). In the case of PVA-based polymeric systems such as the currently fabricated fibers which were highly water-absorbent, the entrance of water into the polymer leads to the formation of a jelly-like system that in part may slow down the release of drug from the deeper parts (32). Another hindering factor of the release is the presence of the shell layer in F_3_ fibers. 

As a part of the biological assay, F_3_ fibers (with or without Dex) were chosen to be exposed to human fibroblasts *in vitro* for biocompatibility assessment. Although both neat and Dex-loaded F_3_ fibers presented good biocompatibility, the latter showed significantly higher viability of cells on day 5. It indicates that Dex probably exerted an independent enhancing effect on fibroblast viability. In this regard, research previously showed that PLLA/PEO hybrid nanofibers loaded with Dex or Dex-Argireline were cytocompatible with human dermal fibroblasts using direct and indirect methods (25). In addition, researchers reported that Dex-loaded PVA-alginate-CS fibers were superior to neat fibers in terms of fibroblast activation and proliferation (26). The biocompatibility findings using the MTT test in the present work were confirmed by fibroblasts’ morphology on the F_3_ fibers as observed in the SEM photos (Figure 6). The Dex-loaded F_3_-treated fibroblast was attached and fully stretched in contrast to the cell on the neat F_3_ fibers. This morphology was previously reported to be associated with an activation state of fibroblasts. These fibroblasts make dendritic processes migrate and spread efficiently on the extracellular matrix (33).

Findings of the wound healing assay in animals confirmed the findings of the *in vitro* study because Dex-loaded F_3_ fibers had a better performance in reducing wound size and improving histologic parameters of wound healing than its neat counterpart. As both Dex-loaded and neat F_3_ fibers shared the same structure, the superiority of Dex-loaded F_3_ fibers in wound healing might be directly attributed to the presence of Dex. Previous studies have shown enhanced wound healing by Dex in both clinical and experimental setups. For example, Gorski *et al*. reported that early topical treatment with Dex was associated with improvement of superficial post-procedure wounds and that gene expression data supported these clinical effects (3). In an animal study on burn wounds, hybrid nanofibers loaded with Dex alone or in combination with another bioactive agent, yielded smaller wounds compared to control treatments after 14 days. They also enhanced angiogenesis, collagen production, and re-epithelization (25). Similarly, another study reported beneficial wound healing effects in mice by an alginate-based core/shell nanofibrous mat loaded with Dex in terms of effects on wound closure (27). To fill in the gap left by other researchers, oxidation-reduction status in wounds treated by Dex-loaded electrospun fibers was examined in the present study. Dex-loaded F_3_ fibers exhibited significant anti-oxidant properties in terms of lowering MDA and elevating SOD levels compared to the neat F_3_ fibers. This finding is consistent with both *in vitro* and *in vivo* wound healing findings by Dex-loaded F_3_ fibers. Hence, it is postulated that the observed beneficial effects on wound healing were in part associated with the reduction in tissue oxidative stress by Dex-loaded F_3_ fibers. The anti-oxidant properties of Dex were also reported in other studies; for example, the anti-oxidant activities of Dex in the kidneys of diabetic rats (4) and in ischemia-reperfusion-induced neuropathy in rabbits (5) were previously reported. In the inflammatory phase of wound healing, pro-inflammatory cytokines and phagocytic immune cells such as macrophages and neutrophils are recruited to the wound site which in turn leads to a rise in tissue oxidative stress. This is quite necessary at the beginning of the wound-healing process but may hinder proper wound healing if persisting in later phases (34). Therefore, it can be presumed that the gradual release of Dex from F_3_ core/shell fibers throughout the healing period (14 days) might have controlled unnecessary tissue oxidative stress leading to probably less oxidative-induced dysfunction in fibroblasts, collagen fibers, etc. (35). 

**Table 1:** Electrospinning parameters for the fabrication of core/shell fibers

**Injection speed (ml/hr) of solution**			Distance (cm)*	**Voltage (kV)**	**Fiber **
**Core**		**Shell**			
0.30	0		12	18	F_1_
0.30	0.15		12	18	F_2_
0.30	0.30		12	18	F_3_

F1: Nanofiber with PVA/Dex in core without shell layer

F2: Nanofiber with PVA/Dex in core and PVA/CS in shell at shell injection rate of 0.15 ml/hr

F3: Nanofiber with PVA/Dex in core and PVA/CS in shell at shell injection rate of 0.30 ml/hr

* Distance from the nozzle to the collector

PVA: polyvinyl alcohol, Dex: dexpanthenol, CS: chitosan

**Table 2:** Release kinetic models used to predict Dex release mechanism from F1 and F3 fibers

Model	General equation	Resolved equation	
		F_1_ fiber	F_3_ fiber
Zero order	$C_{t}/C_{\mathrm{total}}=k_{0}t$	$C_{t}/C_{\mathrm{total}}=0.57t$ R^2^ = 0.53	$C_{t}/C_{\mathrm{total}}=0.42t$ R^2^ = 0.60
First order	$1-(C_{t}/C_{\mathrm{total}})=e^{-k_{1}t}$	$1-(C_{t}/C_{\mathrm{total}})=e^{-0.017t}$ R^2^ = 0.82	$1-(C_{t}/C_{\mathrm{total}})=e^{-0.003t}$ R^2^ = 0.75
Higuchi	$C_{t}/C_{\mathrm{total}}={k_{H}t}^{0.5}$	$C_{t}/C_{\mathrm{total}}={23.22t}^{0.5}$ R^2^ = 0.99	$C_{t}/C_{\mathrm{total}}={14.33t}^{0.5}$ R^2^ = 0.93
Korsmeyer-Peppas	$C_{t}/C_{\mathrm{total}}={k_{P}t}^{n}$	$C_{t}/C_{\mathrm{total}}={1.40t}^{0.49}$ R^2^ = 0.99	$C_{t}/C_{\mathrm{total}}={1.31t}^{0.37}$ R^2^ = 0.94

Ct/Ctotal is the fraction of Dex released at time t relative to the total amount loaded.

k0, k1, kH, and kP: rate constants

n: diffusion exponent

Swelling equilibrium was reached almost after 18 hr for all fibers.

The major characteristic bands of pure materials were marked by the corresponding wave numbers.

FTIR: fourier transform infrared spectroscopy, PVA: polyvinyl alcohol, Dex: dexpanthenol, CS: chitosan

Data are means ± standard deviation of replicated measurements (n=3).

Dex: dexpanthenol

The inset pictures show representative fibroblasts attached either on Dex-loaded F3 fibers (above) or on neat F3 fibers (below) on day 5 of incubation.

* indicates significant difference compared to the neat F3 fibers at the selected day (*P*<0.05)

Dex: dexpanthenol

The graph on the right side shows mean values of wound size on different post-surgical days.

Dex: dexpanthenol

Re-epithelization edges were marked with arrows (→) in H&E stained samples.

E: epithelial layer, C: collagen fibers, GT: granulation tissue.

* significant difference as compared with normal saline (*P*<0.05)

# significant difference as compared with neat F3 fibers (*P*<0.05)

## Conclusion

Modulation of oxidative stress has a determining role in the fate of wound healing. One of the important findings in the present study was a significant decrease in the oxidative stress in tissues treated by Dex-loaded core/shell nanofibers. Such wounds also showed a better healing profile than wounds treated with neat nanofibers. Therefore, we can deduce that the intrinsic anti-oxidant activity of Dex had much influence on the healing process. All in all, the fabricated wound dressing can be considered a new topical drug delivery system that delivers an anti-oxidant drug (Dex) in a sustained manner to the wounded tissue through a nanofibrous scaffold. Hence, the fabricated nanofibers open new horizons for more clinical investigations with emphasis on lowering oxidative stress in the field of wound care. 

## Authors’ Contributions

S Z conceived and designed the study and analyzed the data. SS H, MN, and MM K performed the experiments. SSH and M P prepared the draft manuscript. S Z and RS supervised and edited the article and approved the final version to be published. M A took part in funding acquisition and providing lab facilities. 

## Conflicts of Interest

None.
